# *catena*-Poly[[dianilinedichloridocopper(II)]-μ_2_-2,5-bis­(4-pyrid­yl)-1,3,4-oxadiazole]

**DOI:** 10.1107/S1600536809054191

**Published:** 2009-12-24

**Authors:** Qinglong Meng, Yiming Wu, Chi Zhang

**Affiliations:** a School of Chemistry and Chemical Engineering, Jiangsu University, 301 Xuefu Road, Zhenjiang 212013, Jiangsu, People’s Republic of China; b Research Center for Advanced Molecular Materials, School of Chemistry and Chemical Engineering, Scientific Research Academy, Jiangsu University, 301 Xuefu Road, Zhenjiang 212013, Jiangsu, People’s Republic of China

## Abstract

In the title compound, [CuCl_2_(C_6_H_7_N)_2_(C_12_H_8_N_4_O)]_*n*_, the Cu atom, located on an inversion center, is coordinated by four N atoms from two aniline ligands and two 2,5-bis­(4-pyrid­yl)-1,3,4-oxadiazole ligands. Two Cl atoms lying above and below the plane formed by these four N atoms inter­act weakly with the Cu atom [Cu—Cl = 2.7870 (7) Å]. The *trans* 2,5-bis­(4-pyrid­yl)-1,3,4-oxadiazole ligands act as bridging ligands, linking adjacent Cu atoms and forming a one-dimensional coordination polymer. Two anilines coordinate with each Cu atom as terminal groups. The structure contains two classical N—H⋯Cl and two non-classical C—H⋯Cl hydrogen bonds.

## Related literature

Unsymmetric organic bridging ligands can play different roles in the construction of metal-organic frameworks, see: Du *et al.* (2004 ▶); Dong *et al.* (2005 ▶). For Cu—Cl distances, see: Handley *et al.* (2001 ▶). 
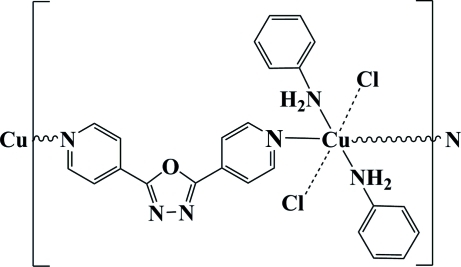


## Experimental

### 

#### Crystal data

[CuCl_2_(C_6_H_7_N)_2_(C_12_H_8_N_4_O)]
*M* *_r_* = 544.93
Monoclinic, 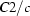

*a* = 27.028 (5) Å
*b* = 12.618 (3) Å
*c* = 6.7904 (14) Å
β = 94.96 (3)°
*V* = 2307.1 (8) Å^3^
*Z* = 4
Mo *K*α radiation
μ = 1.21 mm^−1^
*T* = 293 K
0.20 × 0.20 × 0.20 mm

#### Data collection

Rigaku CCD area-detector diffractometer
Absorption correction: multi-scan (*ABSCOR*; Higashi, 1995 ▶) *T*_min_ = 0.329, *T*_max_ = 0.463
5331 measured reflections
2233 independent reflections
2106 reflections with *I* > 2σ(*I*)
*R*_int_ = 0.018

#### Refinement

*R*[*F* ^2^ > 2σ(*F* ^2^)] = 0.033
*wR*(*F* ^2^) = 0.085
*S* = 1.04
2233 reflections
156 parameters
H-atom parameters constrained
Δρ_max_ = 0.33 e Å^−3^
Δρ_min_ = −0.31 e Å^−3^

### 

Data collection: *CrystalClear* (Rigaku, 2008 ▶); cell refinement: *CrystalClear*; data reduction: *CrystalClear*; program(s) used to solve structure: *SHELXS97* (Sheldrick, 2008 ▶); program(s) used to refine structure: *SHELXL97* (Sheldrick, 2008 ▶); molecular graphics: *SHELXTL* (Sheldrick, 2008 ▶); software used to prepare material for publication: *SHELXTL*.

## Supplementary Material

Crystal structure: contains datablocks I, global. DOI: 10.1107/S1600536809054191/pv2245sup1.cif

Structure factors: contains datablocks I. DOI: 10.1107/S1600536809054191/pv2245Isup2.hkl

Additional supplementary materials:  crystallographic information; 3D view; checkCIF report

## Figures and Tables

**Table 1:** Hydrogen-bond geometry (Å, °)

*D*—H⋯*A*	*D*—H	H⋯*A*	*D*⋯*A*	*D*—H⋯*A*
N1—H1*A*⋯Cl1^i^	0.90	2.53	3.406 (2)	165
N1—H1*B*⋯Cl1^ii^	0.90	2.56	3.393 (2)	154
C9—H9*A*⋯Cl1^iii^	0.93	2.70	3.285 (2)	121
C2—H2*C*⋯Cl1	0.93	2.66	3.328 (2)	129

Symmetry codes: (i) 
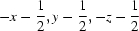
; (ii) 
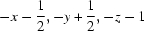
; (iii) 
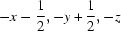
.

**Fig. 1.**
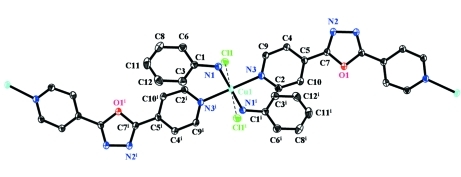
The molecular structure of the title compound, with atom labels and 30% probability displacement ellipsoids; H atoms have been omitted for clarity. Symmetry code: (i) -x - 1/2, -y + 1/2, -z.

## supplementary crystallographic information

### Comment

The unsymmetric organic bridging ligands can play different roles in constructing metal-organic frameworks (Du *et al.*, 2004; Dong *et al.*, 2005). Recently, we have synthesized a new one-dimensional polymer with unsymmetric organic 2,5-bis(4-pyridyl)-1,3,4-oxadiazole as bridging ligand. In this paper, the crystal structure of the title compound, (I), is presented.

As illustrated in Fig. 1, each Cu coordinates with four N atoms from two anilines and two 2,5-bis(4-pyridyl)-1,3,4-oxadiazole ligands, and two Cl atoms lying above and below the plane formed by the N atoms around Cu interact with Cu atom to form an octahedral geometry. The Cu—Cl bonds (2.7870 (7) Å) are longer than normal value (Handley *et al.*, 2001). 2,5-Bis(4-pyridyl)-1,3,4-oxadiazoles act as bridging ligands to connect adjacent two Cu atoms to construct a unique one-dimensional chain. The crystal structure shows a range of classical N—H···Cl and non-classical C—H···Cl hydrogen bonds (Table 1).

### Experimental

2,5-Bis(4-pyridyl)-1,3,4-oxadiazole (1 mmol) and copper chloride (1 mmol) were added into *N*,*N*'-dimethylformamide (5 ml) with thorough stirring for 5 minutes. The solution underwent an additional stir for one minute after aniline (2 ml) was added. After filtration, 10 ml i-PrOH was successively laid on the surface of above filtrate. Black block crystals were obtained after ten days.

### Refinement

H atoms were positioned geometrically and refined with riding model, with C—H = 0.93 Å and N—H = 0.90 Å and *U*_iso_ = 1.2*U*_eq_(parent atom) for all H atoms.

### Crystal data

**Table:**

[CuCl_2_(C_6_H_7_N)_2_(C_12_H_8_N_4_O)]	*F*(000) = 1116
*M**_r_* = 544.93	*D*_x_ = 1.569 Mg m^−^^3^
Monoclinic, *C*2/*c*	Mo *K*α radiation, λ = 0.71073 Å
Hall symbol: -C 2yc	Cell parameters from 4915 reflections
*a* = 27.028 (5) Å	θ = 3.0–28.9°
*b* = 12.618 (3) Å	µ = 1.21 mm^−^^1^
*c* = 6.7904 (14) Å	*T* = 293 K
β = 94.96 (3)°	Prism, black
*V* = 2307.1 (8) Å^3^	0.20 × 0.20 × 0.20 mm
*Z* = 4	

### Data collection

**Table:**

Rigaku CCD area-detector diffractometer	2233 independent reflections
Radiation source: fine-focus sealed tube	2106 reflections with *I* > 2σ(*I*)
graphite	*R*_int_ = 0.018
Detector resolution: 28.5714 pixels mm^-1^	θ_max_ = 26.0°, θ_min_ = 3.0°
phi and ω scans	*h* = −28→33
Absorption correction: multi-scan (*ABSCOR*; Higashi, 1995)	*k* = −15→13
*T*_min_ = 0.329, *T*_max_ = 0.463	*l* = −8→8
5331 measured reflections	

### Refinement

**Table:**

Refinement on *F*^2^	Primary atom site location: structure-invariant direct methods
Least-squares matrix: full	Secondary atom site location: difference Fourier map
*R*[*F*^2^ > 2σ(*F*^2^)] = 0.033	Hydrogen site location: inferred from neighbouring sites
*wR*(*F*^2^) = 0.085	H-atom parameters constrained
*S* = 1.03	*w* = 1/[σ^2^(*F*_o_^2^) + (0.0436*P*)^2^ + 2.9828*P*] where *P* = (*F*_o_^2^ + 2*F*_c_^2^)/3
2233 reflections	(Δ/σ)_max_ < 0.001
156 parameters	Δρ_max_ = 0.33 e Å^−^^3^
0 restraints	Δρ_min_ = −0.31 e Å^−^^3^

### Special details

**Table:**

Geometry. All e.s.d.'s (except the e.s.d. in the dihedral angle between two l.s. planes) are estimated using the full covariance matrix. The cell e.s.d.'s are taken into account individually in the estimation of e.s.d.'s in distances, angles and torsion angles; correlations between e.s.d.'s in cell parameters are only used when they are defined by crystal symmetry. An approximate (isotropic) treatment of cell e.s.d.'s is used for estimating e.s.d.'s involving l.s. planes.
Refinement. Refinement of *F*^2^ against ALL reflections. The weighted *R*-factor *wR* and goodness of fit *S* are based on *F*^2^, conventional *R*-factors *R* are based on *F*, with *F* set to zero for negative *F*^2^. The threshold expression of *F*^2^ > σ(*F*^2^) is used only for calculating *R*-factors(gt) *etc*. and is not relevant to the choice of reflections for refinement. *R*-factors based on *F*^2^ are statistically about twice as large as those based on *F*, and *R*- factors based on ALL data will be even larger.

### Fractional atomic coordinates and isotropic or equivalent isotropic displacement parameters (Å^2^)

**Table:**

	*x*	*y*	*z*	*U*_iso_*/*U*_eq_	
Cu1	−0.2500	0.2500	0.0000	0.03226 (14)	
Cl1	−0.25356 (2)	0.38364 (4)	−0.32770 (8)	0.03830 (16)	
O1	−0.5000	0.13053 (16)	−0.2500	0.0314 (4)	
N1	−0.21455 (6)	0.14463 (14)	−0.1808 (3)	0.0307 (4)	
H1A	−0.2260	0.0793	−0.1575	0.037*	
H1B	−0.2248	0.1606	−0.3069	0.037*	
N2	−0.47412 (6)	−0.03453 (15)	−0.2314 (3)	0.0439 (5)	
N3	−0.31537 (6)	0.18313 (14)	−0.1116 (2)	0.0302 (4)	
C1	−0.16136 (8)	0.13839 (16)	−0.1674 (3)	0.0297 (4)	
C2	−0.35055 (8)	0.24122 (16)	−0.2121 (3)	0.0318 (5)	
H2C	−0.3421	0.3077	−0.2578	0.038*	
C3	−0.13423 (9)	0.21652 (19)	−0.2524 (3)	0.0384 (5)	
H3A	−0.1504	0.2698	−0.3277	0.046*	
C4	−0.37354 (7)	0.04244 (17)	−0.0989 (3)	0.0327 (5)	
H4A	−0.3801	−0.0273	−0.0650	0.039*	
C5	−0.41057 (7)	0.10551 (16)	−0.1900 (3)	0.0294 (4)	
C6	−0.13728 (9)	0.05767 (19)	−0.0619 (3)	0.0393 (5)	
H6A	−0.1554	0.0039	−0.0081	0.047*	
C7	−0.46097 (7)	0.06337 (17)	−0.2226 (3)	0.0316 (4)	
C8	−0.08592 (10)	0.0568 (2)	−0.0361 (4)	0.0526 (7)	
H8A	−0.0696	0.0023	0.0355	0.063*	
C9	−0.32679 (7)	0.08464 (17)	−0.0592 (3)	0.0315 (4)	
H9A	−0.3022	0.0430	0.0065	0.038*	
C10	−0.39873 (8)	0.20621 (17)	−0.2503 (3)	0.0324 (4)	
H10A	−0.4227	0.2495	−0.3153	0.039*	
C11	−0.05886 (9)	0.1359 (2)	−0.1157 (4)	0.0546 (7)	
H11A	−0.0244	0.1357	−0.0958	0.066*	
C12	−0.08290 (9)	0.2150 (2)	−0.2247 (4)	0.0489 (6)	
H12A	−0.0646	0.2678	−0.2804	0.059*	

### Atomic displacement parameters (Å^2^)

**Table:**

	*U*^11^	*U*^22^	*U*^33^	*U*^12^	*U*^13^	*U*^23^
Cu1	0.0197 (2)	0.0341 (2)	0.0427 (2)	−0.00195 (14)	0.00072 (15)	−0.00980 (15)
Cl1	0.0389 (3)	0.0335 (3)	0.0414 (3)	−0.0028 (2)	−0.0027 (2)	0.0045 (2)
O1	0.0211 (9)	0.0319 (10)	0.0406 (11)	0.000	−0.0006 (8)	0.000
N1	0.0295 (9)	0.0312 (9)	0.0313 (9)	−0.0004 (7)	0.0024 (7)	0.0004 (7)
N2	0.0222 (9)	0.0345 (10)	0.0735 (14)	0.0003 (8)	−0.0040 (9)	0.0004 (9)
N3	0.0229 (8)	0.0341 (9)	0.0336 (9)	0.0001 (7)	0.0016 (7)	−0.0058 (7)
C1	0.0298 (10)	0.0310 (10)	0.0287 (10)	0.0015 (8)	0.0055 (8)	−0.0039 (8)
C2	0.0296 (11)	0.0328 (11)	0.0331 (11)	−0.0014 (8)	0.0034 (9)	0.0006 (8)
C3	0.0409 (13)	0.0385 (12)	0.0368 (12)	−0.0018 (10)	0.0094 (10)	0.0013 (9)
C4	0.0268 (10)	0.0313 (10)	0.0395 (11)	0.0012 (9)	0.0004 (8)	−0.0019 (9)
C5	0.0225 (10)	0.0345 (11)	0.0310 (10)	−0.0015 (8)	0.0010 (8)	−0.0046 (8)
C6	0.0396 (12)	0.0385 (12)	0.0405 (12)	0.0028 (10)	0.0072 (10)	0.0022 (10)
C7	0.0230 (9)	0.0337 (11)	0.0375 (11)	0.0034 (9)	−0.0005 (8)	−0.0008 (9)
C8	0.0423 (14)	0.0611 (17)	0.0538 (15)	0.0199 (13)	0.0007 (11)	0.0028 (13)
C9	0.0245 (10)	0.0323 (10)	0.0371 (11)	0.0027 (9)	−0.0009 (8)	−0.0031 (9)
C10	0.0261 (10)	0.0364 (11)	0.0341 (11)	0.0035 (9)	−0.0018 (8)	0.0016 (9)
C11	0.0289 (12)	0.0780 (19)	0.0577 (16)	0.0022 (13)	0.0085 (11)	−0.0111 (14)
C12	0.0421 (13)	0.0549 (15)	0.0522 (15)	−0.0115 (12)	0.0191 (12)	−0.0065 (12)

### Geometric parameters (Å, °)

**Table:**

Cu1—N3^i^	2.0436 (17)	C2—H2C	0.9300
Cu1—N3	2.0436 (17)	C3—C12	1.384 (3)
Cu1—N1^i^	2.0966 (17)	C3—H3A	0.9300
Cu1—N1	2.0966 (17)	C4—C9	1.376 (3)
Cu1—Cl1	2.7870 (7)	C4—C5	1.383 (3)
Cu1—Cl1^i^	2.7870 (7)	C4—H4A	0.9300
O1—C7	1.353 (2)	C5—C10	1.381 (3)
O1—C7^ii^	1.353 (2)	C5—C7	1.461 (3)
N1—C1	1.435 (3)	C6—C8	1.384 (3)
N1—H1A	0.9000	C6—H6A	0.9300
N1—H1B	0.9000	C8—C11	1.375 (4)
N2—C7	1.285 (3)	C8—H8A	0.9300
N2—N2^ii^	1.400 (3)	C9—H9A	0.9300
N3—C9	1.336 (3)	C10—H10A	0.9300
N3—C2	1.340 (3)	C11—C12	1.372 (4)
C1—C6	1.376 (3)	C11—H11A	0.9300
C1—C3	1.384 (3)	C12—H12A	0.9300
C2—C10	1.378 (3)		
			
N3^i^—Cu1—N3	180.00	C10—C2—H2C	118.7
N3^i^—Cu1—N1^i^	86.87 (7)	C12—C3—C1	119.6 (2)
N3—Cu1—N1^i^	93.13 (7)	C12—C3—H3A	120.2
N3^i^—Cu1—N1	93.13 (7)	C1—C3—H3A	120.2
N3—Cu1—N1	86.87 (7)	C9—C4—C5	118.8 (2)
N1^i^—Cu1—N1	180.00	C9—C4—H4A	120.6
N3^i^—Cu1—Cl1	90.87 (6)	C5—C4—H4A	120.6
N3—Cu1—Cl1	89.13 (6)	C10—C5—C4	119.00 (19)
N1^i^—Cu1—Cl1	95.61 (5)	C10—C5—C7	121.81 (19)
N1—Cu1—Cl1	84.39 (5)	C4—C5—C7	119.19 (19)
N3^i^—Cu1—Cl1^i^	89.13 (6)	C1—C6—C8	119.7 (2)
N3—Cu1—Cl1^i^	90.87 (6)	C1—C6—H6A	120.1
N1^i^—Cu1—Cl1^i^	84.39 (5)	C8—C6—H6A	120.1
N1—Cu1—Cl1^i^	95.61 (5)	N2—C7—O1	112.73 (18)
Cl1—Cu1—Cl1^i^	180.00	N2—C7—C5	127.37 (19)
C7—O1—C7^ii^	102.5 (2)	O1—C7—C5	119.89 (18)
C1—N1—Cu1	120.26 (13)	C11—C8—C6	120.4 (2)
C1—N1—H1A	107.3	C11—C8—H8A	119.8
Cu1—N1—H1A	107.3	C6—C8—H8A	119.8
C1—N1—H1B	107.3	N3—C9—C4	122.48 (19)
Cu1—N1—H1B	107.3	N3—C9—H9A	118.8
H1A—N1—H1B	106.9	C4—C9—H9A	118.8
C7—N2—N2^ii^	106.03 (12)	C2—C10—C5	118.60 (19)
C9—N3—C2	118.33 (18)	C2—C10—H10A	120.7
C9—N3—Cu1	119.83 (14)	C5—C10—H10A	120.7
C2—N3—Cu1	121.05 (14)	C12—C11—C8	119.8 (2)
C6—C1—C3	120.0 (2)	C12—C11—H11A	120.1
C6—C1—N1	119.99 (19)	C8—C11—H11A	120.1
C3—C1—N1	119.90 (19)	C11—C12—C3	120.4 (2)
N3—C2—C10	122.5 (2)	C11—C12—H12A	119.8
N3—C2—H2C	118.7	C3—C12—H12A	119.8

Symmetry codes: (i) −*x*−1/2, −*y*+1/2, −*z*; (ii) −*x*−1, *y*, −*z*−1/2.

### Hydrogen-bond geometry (Å, °)

**Table:**

*D*—H···*A*	*D*—H	H···*A*	*D*···*A*	*D*—H···*A*
N1—H1A···Cl1^iii^	0.90	2.53	3.406 (2)	165
N1—H1B···Cl1^iv^	0.90	2.56	3.393 (2)	154
C9—H9A···Cl1^i^	0.93	2.70	3.285 (2)	121
C2—H2C···Cl1	0.93	2.66	3.328 (2)	129

Symmetry codes: (iii) −*x*−1/2, *y*−1/2, −*z*−1/2; (iv) −*x*−1/2, −*y*+1/2, −*z*−1; (i) −*x*−1/2, −*y*+1/2, −*z*.
